# A prediction model for neonatal necrotizing enterocolitis in preterm and very low birth weight infants

**DOI:** 10.3389/fped.2023.1242978

**Published:** 2023-10-18

**Authors:** Baoying Feng, Zhihui Zhang, Qiufen Wei, Yan Mo, Mengmeng Luo, Lianfang Jing, Yan Li

**Affiliations:** ^1^ Maternal and Child Health Hospital of Guangxi Zhuang Autonomous Region, Nanning, China; ^2^ Guangxi Clinical Research Center for Pediatric Disease, Nanning, China; ^3^ Department of Applied Mathematics, The Hong Kong Polytechnic University, Kowloon, Hong Kong SAR, China; ^4^ Department of Biological Sciences, University of Liverpool, Liverpool, United Kingdom

**Keywords:** necrotizing enterocolitis, preterm infant, low birth weight, risk factor, principal component analysis

## Abstract

**Objectives:**

Neonatal necrotizing enterocolitis (NEC) is a severe gastrointestinal disease that primarily affects preterm and very low birth weight infants, with high morbidity and mortality. We aim to build a reliable prediction model to predict the risk of NEC in preterm and very low birth weight infants.

**Methods:**

We conducted a retrospective analysis of medical data from infants (gestational age <32 weeks, birth weight <1,500 g) admitted to Maternal and Child Health Hospital of Guangxi Zhuang Autonomous Region. We collected clinical data, randomly dividing it into an 8:2 ratio for training and testing. Multivariate logistic regression was employed to identify significant predictors for NEC. Principal component analysis was used for dimensionality reduction of numerical variables. The prediction model was constructed through logistic regression, incorporating all relevant variables. Subsequently, we calculated performance evaluation metrics, including Receiver Operating Characteristic (ROC) curves and confusion matrices. Additionally, we conducted model performance comparisons with common machine learning models to establish its superiority.

**Results:**

A total of 292 infants were included, with 20% (*n* = 58) randomly selected for external validation. Multivariate logistic regression revealed the significance of four predictors for NEC in preterm and very low birth weight infants: temperature (*P* = 0.003), Apgar score at 5 min (*P* = 0.004), formula feeding (*P* = 0.007), and gestational diabetes mellitus (GDM, *P* = 0.033). The model achieved an accuracy of 82.46% in the test set with an F1 score of 0.90, outperforming other machine learning models (support vector machine, random forest).

**Conclusions:**

Our logistic regression model effectively predicts NEC risk in preterm and very low birth weight infants, as confirmed by external validation. Key predictors include temperature, Apgar score at 5 min, formula feeding, and GDM. This study provides a vital tool for NEC risk assessment in this population, potentially improving early interventions and child survival. However, clinical validation and further research are necessary for practical application.

## 1. Introduction

Neonatal necrotizing enterocolitis (NEC), defined as a serious neonatal inflammatory bowel disease, is the most common and lethal gastrointestinal tract disease in the neonates (1). NEC is most often seen in preterm infants, especially those born at <32 weeks of gestation. It has also been shown that the incidence of NEC is inversely correlated with gestational age (2, 3). Previous studies have shown that the incidence of NEC of Bell's stage 2 or higher in extremely premature ranges from 1.7% in Japan to 6.9% in Spain (4–6). A third of infants with birth weight <1,500 g or who born <32 weeks of gestation are referred for laparotomies; this percentage rises to 60% in the most immature infants (6, 7). Overall, the mortality rate is about one third, but it is as high as 50% for infants who undergo laparotomy (6, 7). Several short-term or long-term complications may occur in NEC newborns, such as poor growth, short bowel syndrome, and poorer neuro-developmental outcomes (8–11). NEC is a disease caused by a combination of factors. Its etiology and pathogenesis are still not fully understood. Many potential risk factors have been associated with its development. At present, some factors affecting NEC have been reported, such as premature delivery, feeding, infection, antibiotic exposure, patent ductus arteriosus, hypoxia and so on (1, 12–15). However, among 64 identified risk factors for NEC, a panel of 35 international experts reached a high level of consensus only on gestational age, birth weight, and feeding (16). Therefore, it is important to identify the risk factors of NEC for clinical prevention and treatment. The risk factors for NEC are now the subject of a small number of studies, but there are few reports on how to achieve individualized NEC prediction, and it is even more uncommon to apply individualized prediction models in clinical diagnosis and therapy.

In summary, NEC is a life-threatening intestinal inflammation that typically occurs in preterm or low birth weight infants (17). Until now, there has been a lack of practical clinical prediction models for NEC. The aim of this study was to establish a clinical prediction model for NEC in newborns with gestational age less than 32 weeks and birth weight less than 1,500 g, and to externally validate its accuracy in predicting the risk of NEC. The prediction model will provide clinicians with an early predictive tool for NEC and timely preventive interventions for high-risk infants, helping to reduce the incidence of NEC in preterm and very low birth weight infants and improve the quality of life of newborns in the region.

## 2. Materials and methods

### 2.1. Study population

The present retrospective study was performed by reviewing infant's medical records. It was approved by the Medical Ethics Committee of Maternal and Child Health Hospital of Guangxi Zhuang Autonomous Region. Informed consent was waived considering the retrospective study design.

The present retrospective study was performed by reviewing infant's medical records. We conducted a retrospective study of infants admitted to the Department of Neonatology of Maternity and Child Health Care of Guangxi Zhuang Autonomous Region between September 2019 and September 2021. Our inclusion criteria were as follows: (1) infants <32 weeks of gestation and birth weight <1,500 g; (2) transferred to the Department of Neonatology within 24 h after birth. From September 2019 to September 2021, a total of 308 infants were enrolled. In this study, we excluded 11 infants with birth defects, and 5 infants missing information. Finally, 292 infants were included in the study. This sample was used to build the prediction model.

### 2.2. Data collection

Clinical information of selected infants was collected from the electronic medical record system and included the clinical information on data within 24 h after birth (gestational age, birth weight, head circumference, temperature, sex, Apgar score at 5 min, number of births, mode of delivery, feeding and probiotic administration), as well as maternal perinatal information [maternal age, regular antenatal care, parity, gestational diabetes mellitus (GDM), hypertensive disorders of pregnancy (HDP), maternal infection, premature rupture of membrane (PROM), chorioamnionitis, antenatal intervention, spontaneous preterm birth, antepartum hemorrhage, intrauterine growth restriction (IUGR)].

In this study, we diagnosed NEC in newborns using clinical and radiological examination results and classified its severity according to the modified Bell's criteria. Only infants with NEC at stage 2 or above according to Bell's staging were included in this study (18). The Apgar score at 5 min was measured on a scale from 1 to 10, at 5 min after delivery. Formula feeding was defined as fed with formula milk and but not breast milk. Infant's temperature was measured on admission to the neonatal department, and temperatures were measured in degrees Celsius (°C).

### 2.3. Model building

Random assignment was conducted following an 8:2 ratio based on international standard training and test sets. The validation set is not processed in any way and is only used for the validation of the experimental results, and for the training set, we perform logistic regression using all variables.

To develop and evaluate predictive models for NEC in preterm and very low birth weight infants, all potential factors were first analyzed using univariate logistic regression to determine their individual significance in relation to NEC. In previous studies, any factor found to be significant or associated with NEC was included in multivariate logistic regression models to identify independent predictors of NEC. Furthermore, for comprehensive information extraction from the data, all variables were initially considered during predictive modeling. Recognizing that some variables may have a minimal mathematical impact on NEC but are not devoid of influence, we applied principal component analysis to extract valid information while reducing dimensionality. This approach aimed to minimize factor redundancy in model prediction.

Let the multivariate sample of *p* features be denoted as:

$$X=(X_{1},X_{1}\ldots X_{p}),$$

Multiple regression model form:

$$F_{i}=\mu_{1i}X_{i}+\mu_{2i}X_{i}+\ldots +\mu_{\;pi}X_{p},$$

where: $\mu_{i}=(\mu_{1i},\mu_{2i},\ldots ,\mu_{\;pi})$.

If $F_{i}$ satisfies: $F_{i}$ and $F_{j}$ are uncorrelated (orthogonal), and:

$$D(F_{1})\geq D(F_{2})\geq \ldots \geq D(F_{p}),$$

then $F_{i}$ is said to be the *i*-th principal component of the overall $X=(X_{1},X_{1},\ldots ,X_{p})$, of the *i*-th principal component. With *n* samples, each with *p* indicator variables, the sample can be represented as $X=(X_{1},X_{1},\ldots ,X_{p})$.

To normalize the original data:

$$x_{ij}=\frac{x_{ij}-{\bar{x}}_{j}}{\sqrt{\mathrm{var}\;(x_{i})}},$$

where: $\sqrt{\mathrm{var}\;(x_{i})}$ and ${\bar{x}}_{j}$ are the standard deviation and mean of the *j-*th variable, respectively.

Using the standardized data, the standardized matrix is obtained as follows:

$$Z=\left\lceil \begin{array}{ccc} z_{11} & \ldots & z_{1p}\\ \ldots & \ldots & \ldots \\ z_{n1} & \ldots & z_{np} \end{array}\right\rceil ,$$

Calculate the correlation coefficients of the sample matrices:

$$R=[r_{ij}{]}_{\;p\times p}=\frac{Z^{\prime }Z}{n-1},$$

Solve the characteristic equation for the sample correlation coefficient matrix *R* to obtain *P* eigenvalues:

$$\lambda_{1}\geq \lambda_{2}\ldots \geq \lambda_{p},$$

The final result of the principal component analysis equation was obtained as follows:

Y=UX.

Taking the probability of a positive NEC as *P*, the logistic regression model between it and the independent variable is

$$P=\frac{\exp(\beta_{0}+\beta_{1}X_{1}+\ldots +\beta_{k}X_{k})}{1+(\beta_{0}+\beta_{1}X_{1}+\ldots +\beta_{k}X_{k})},$$

The probability of the relative negativity is:

$$1-P=\frac{1}{1+(\beta_{0}+\beta_{1}X_{1}+\ldots +\beta_{k}X_{k})},$$

In order to make the best prediction of the model, the log-likelihood function is constructed and the maximum value of the function is sought by finding the first-order partial derivative to obtain the estimated value of the parameters.

$$L(\beta )=\underset{i}{\Sigma }\{y_{i}\ln[p(x_{i},\beta )]+(1-y_{i})\ln[1-p(x_{i},\beta )]\}$$

### 2.4. Statistical analysis

Categorical variables were reported as a percentage, and continuous data as means and standard deviations (SD). A two-tailed value of *P* < 0.05 was considered statistically significant. *R* and the MATLAB classification model tool were used to predict the sample. Through MATLAB code, including confusion chart, stats of measure, etc., calculate the *F* 1 score, confusion matrix, Area Under Curve (AUC) and importance level of each indicator.

## 3. Results

### 3.1. General characteristics

**Figure 1:**
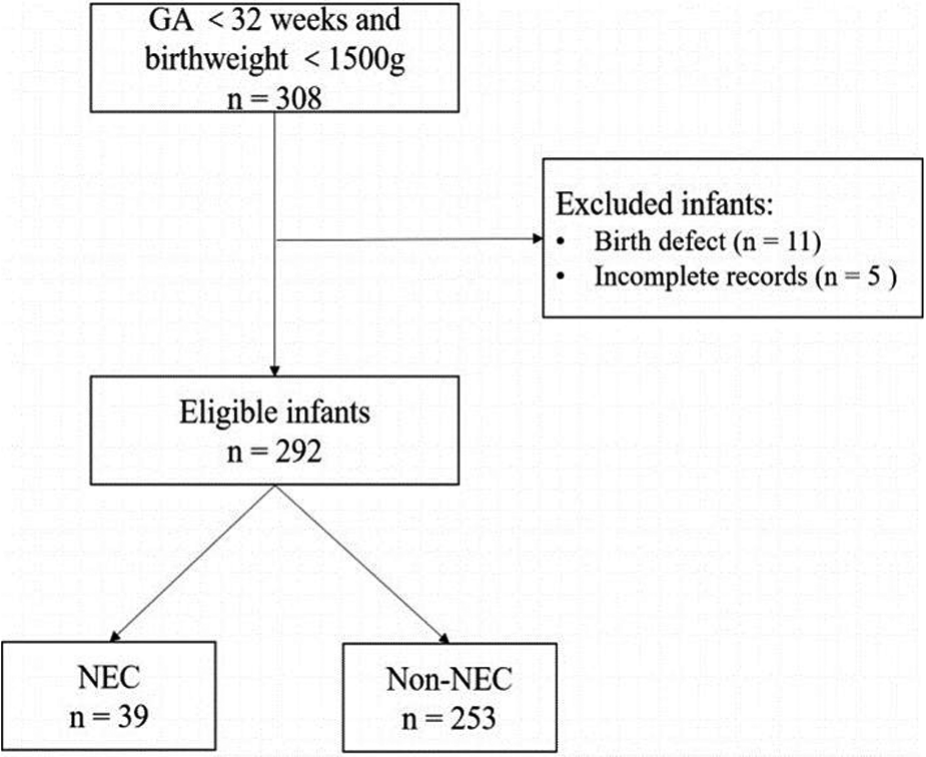
Flow chart for infants’ selection.

In our study, 292 infants were enrolled in this study (Figure 1). The observed incidence of NEC in our study was determined to be 13.3%. Among the entirety of the 292 infants under investigation, a notable subset of 39 infants was identified to have encountered NEC. The mean gestational age of enrolled infants was 28.20 ± 2.01 weeks, with a mean birth weight of 1,087.64 ± 250.72 g. Boys comprised 59.20% of infants. The mean temperature of the infants was 35.66 ± 0.89°C. The mean maternal age at delivery was 31.52 ± 5.30 years old with a range from 15 to 45 years old. The prevalence of GDM and HDP was 22.9% and 13.7%, respectively. The basic demographic and clinical characteristics of participants are presented in Table 1.

**Table 1:** Characteristics of non-NEC vs. NEC group and univariable analysis of risk of NEC.

Variables	Total	Non-NEC	NEC	OR (95% CI)	*P*
Birth weight	292	1,079.2 ± 254.40	1,142.6 ± 220.30	1.001 (1.00–1.002)	0.143
Gestational age	292	28.2 ± 2.10	28.7 ± 1.60	1.12 (0.94–1.34)	0.215
Head circumference	292	26.1 ± 2.60	26.5 ± 2.60	1.06 (0.94–1.2)	0.355
Temperature	292	36.72 ± 0.66	35.20 ± 1.71	0.52 (0.3–0.88)	0.016*
Sex					
Male	173	146 (57.7)	27 (69.2)	0.61 (0.29–1.25)	0.176
Female	119	107 (42.3)	12 (30.8)	1	
Apgar score at 5 min					
0–7 points	21	16 (6.3)	5 (12.8)	1	
8–10 points	271	237 (93.7)	34 (87.2)	2.18 (0.75–6.33)	0.153
Number of births					
Single birth	149	129 (51)	20 (51.3)	1	
Multiple births	143	124 (49)	19 (48.7)	0.99 (0.5–1.94)	0.973
Mode of delivery					
Vaginal delivery	144	129 (51)	15 (38.5)	1	
Caesarean section	148	124 (49)	24 (61.5)	1.72 (0.86–3.43)	0.125
Formula feeding					
No	125	117 (46.2)	8 (20.5)	1	
Yes	167	136 (53.8)	31 (79.5)	3.33 (1.48–7.54)	0.004*
Probiotics					
No	126	107 (42.3)	19 (48.7)	1	
Yes	166	146 (57.7)	20 (51.3)	0.77 (0.39–1.52)	0.452
Maternal age	292	31.53 ± 5.38	31.41 ± 4.86	1 (0.93–1.06)	0.892
Regular antenatal care					
No	5	4 (1.6)	1 (2.6)	1	
Yes	287	249 (98.4)	38 (97.4)	0.59 (0.06–5.38)	0.636
Parity					
Primipara	91	77 (35.4)	14 (35.9)	1	
Multipartum	201	176 (69.6)	25 (64.1)	0.78 (0.39–1.58)	0.494
GDM					
No	225	200 (79.1)	25 (64.1)	1	
Yes	67	53 (20.9)	14 (35.9)	2.11 (1.03–4.35)	0.042*
HDP					
No	252	216 (85.4)	36 (92.3)	1	
Yes	40	37 (14.6)	3 (7.7)	0.49 (0.14–1.66)	0.250
Maternal infection					
No	243	210 (83)	33 (84.6)	1	
Yes	49	43 (17)	6 (15.4)	0.89 (0.35–2.25)	0.802
PROM					
No	208	182 (71.9)	26 (66.7)	1	
Yes	84	71 (28.1)	13 (33.3)	1.28 (0.62–2.63)	0.499
Chorioamnionitis					
No	254	218 (86.2)	36 (92.3)	1	
Yes	38	35 (13.8)	3 (7.7)	0.52 (0.15–1.78)	0.296
Antenatal intervention					
No	271	236 (93.3)	35 (89.7)	1	
Yes	21	17 (6.7)	4 (10.3)	1.6 (0.51–5.03)	0.423
Spontaneous preterm birth					
No	277	239 (94.5)	38 (97.4)	1	
Yes	15	14 (5.5)	1 (2.6)	0.4 (0.06–3.52)	0.446
Antepartum hemorrhage					
No	239	204 (80.6)	35 (89.7)	1	
Yes	53	49 (19.4)	4 (10.3)	0.48 (0.16–1.4)	0.178
Placental abruption					
No	252	217 (85.8)	35 (89.7)	1	
Yes	40	36 (14.2)	4 (10.3)	0.69 (0.23–2.05)	0.504
IUGR					
No	266	230 (90.9)	36 (92.3)	1	
Yes	26	23 (9.1)	3 (7.7)	0.83 (0.24–2.92)	0.776

Values are *n*, *n* (%), or mean ± SD. GA, gestational age; GDM, gestational diabetes mellitus; HDP, hypertensive disorders of pregnancy; PROM, premature rupture of membrane; IUGR, intrauterine growth restriction; OR, odds ratio; CI, confidence interval.

* *P* < 0.05.

### 3.2. Correlation analysis

**Figure 2:**
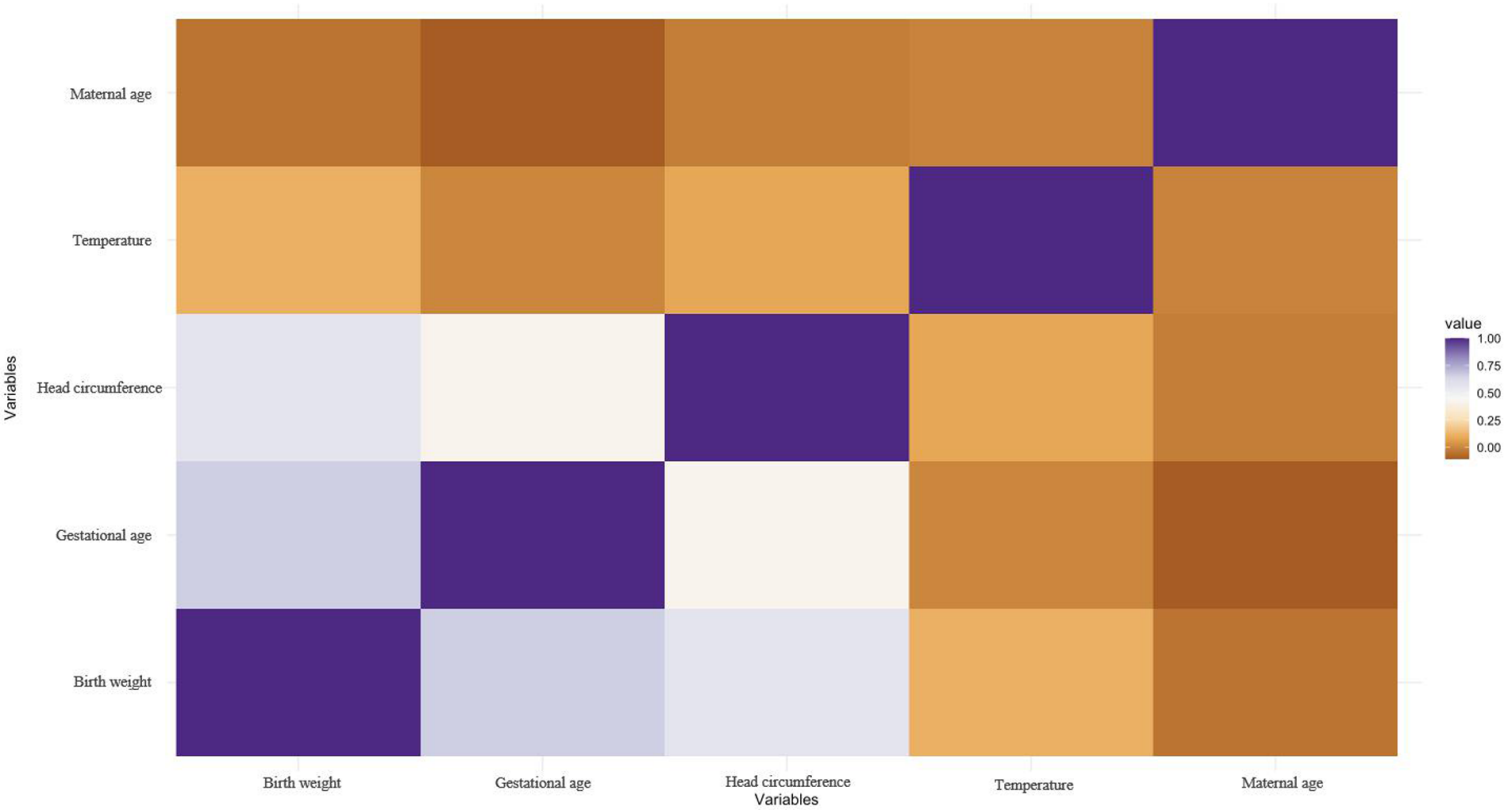
The correlation graph for numeric variables. The correlation graph describes the correlation between several variables, namely gestational age weeks, birth weight, head circumference, maternal age, and temperature.

The correlation graph describes the correlation between several variables, namely gestational age weeks, birth weight, head circumference, maternal age, and temperature. According to (Figure 2), there is some degree of correlation between gestational weeks, birth weight and head circumference. This means that as one variable increases, the likelihood of another variable increasing or decreasing increases. These correlations may be positive, indicating that these variables move in the same direction, or negative, indicating that they move in the opposite direction.

However, the correlations between maternal age and temperature and the other three variables were weak. This suggests that changes in maternal age or temperature do not have a significant effect on fetal or neonatal gestational age, birth weight or head circumference. It is important to note that correlation does not necessarily imply causation. The fact that two variables are correlated does not mean that one variable causes a change in the other variable. There may be other factors contributing to the observed correlation that require further investigation to determine causality. Nonetheless, understanding the associations between these variables is useful for predicting outcomes, identifying risk factors, and informing medical decisions.

### 3.3. Dimensionality reduction

To reduce the dimensionality of the numerical variables, we performed a principal component analysis with the criterion of a maximum variance explained of 75% or more. This resulted in a reduction of the numerical variables to three dimensions.

**Figure 3:**
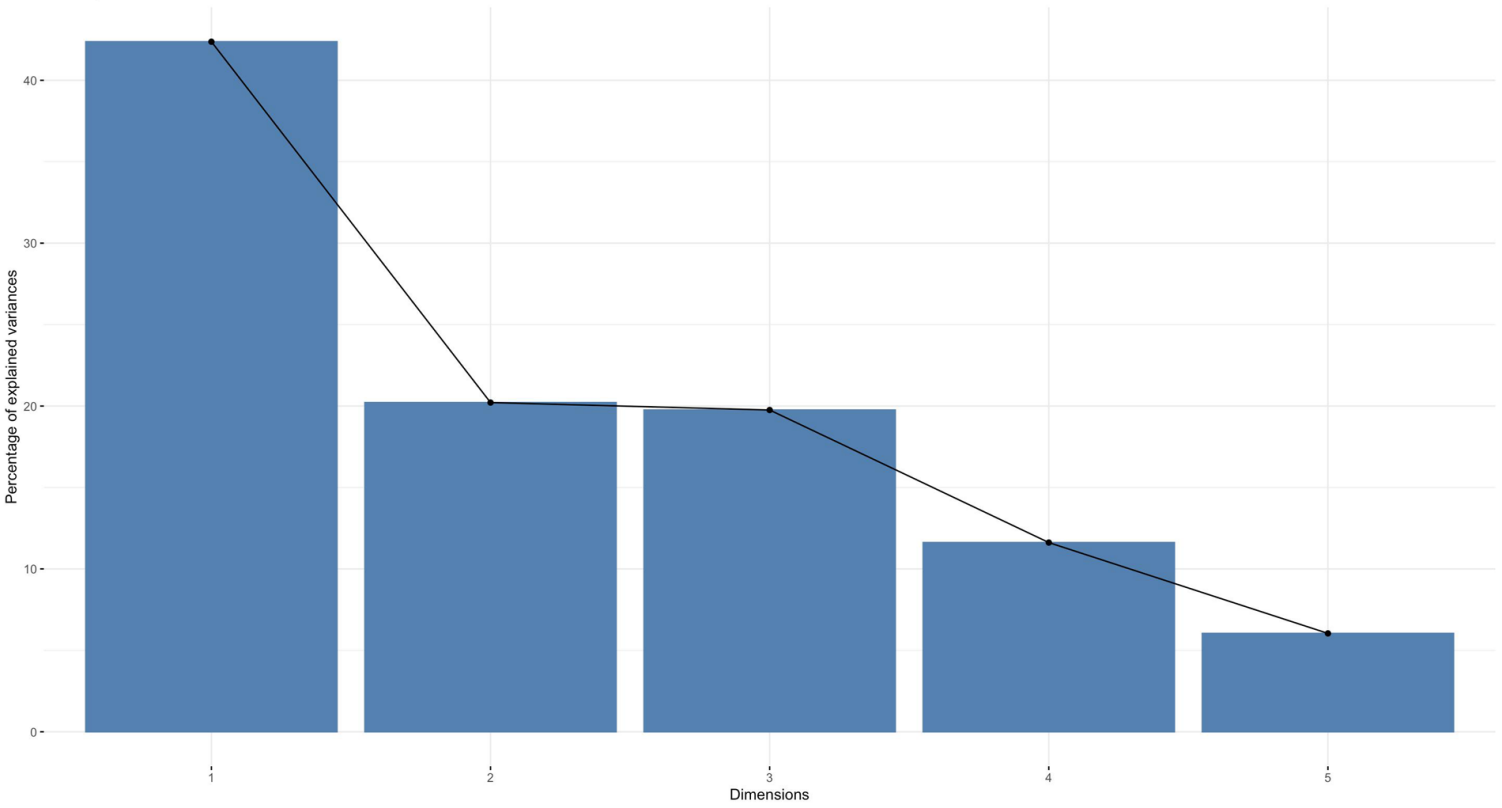
A principal component analysis. A principal component analysis was performed on the numeric variables (principal component analysis explained variance by dimension).

The dimensions are represented by axes, with the length of the axes representing the variance explained. The first dimension has more than 40% of the variance explained and the second dimension has more than 20%. As shown in Figure 3, the first three dimensions combined explain more than 75% of the variance.

**Figure 4:**
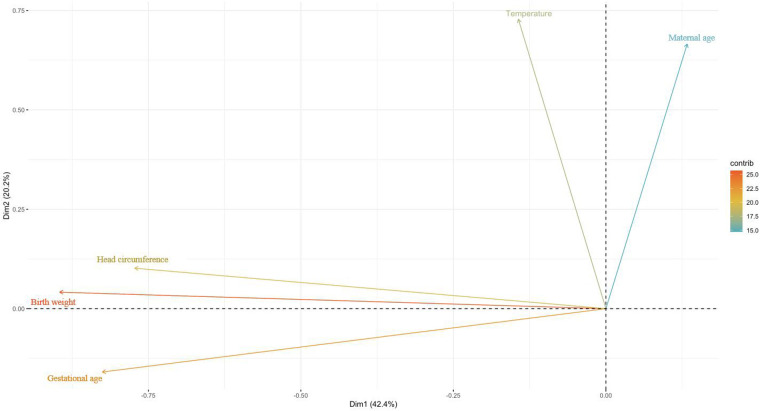
Mapping of numeric variables in two dimensions. Using the maximum explained variance as a criterion, the coordinate axes is the individual dimensions and the length is the explained variance.

The numerical variables were then plotted on a two-dimensional plane. As shown in Figure 4, gestational weeks, birth weight, and head circumference were plotted primarily on the horizontal dimension, while maternal age and body temperature were plotted on the vertical dimension.

**Figure 5:**
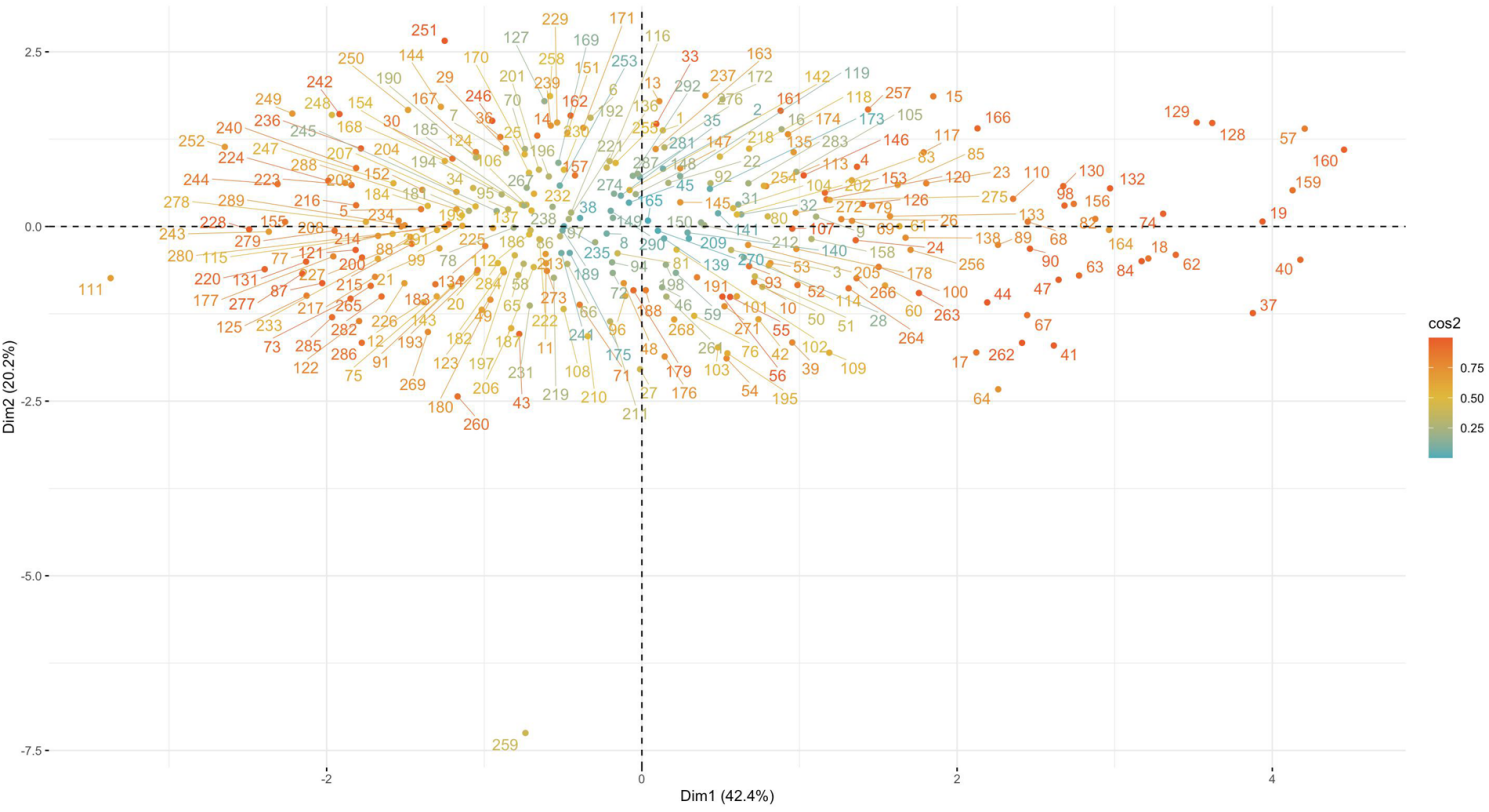
Mapping of samples in two dimensions. Using the maximum explained variance as a criterion, the coordinate axes is the individual dimensions. It can be seen that the cosine value of the sample points in the middle is close to 0, the cosine value of the surrounding sample points is close to 1.

Mapping the sample points on the two-dimensional space consisting of the first and second principal components, it can be seen that the cosine value of the sample points in the middle is close to 0, which is closer to the horizontal plane; meanwhile, the cosine value of the surrounding sample points is close to 1, which is closer to the vertical plane. In addition, the sample points contain some extreme values (Figure 5).

### 3.4. Model tuning

The model was trained and tested using two separate datasets. The training data represents 80% of the total sample and we derived the model results based on this dataset. To select the most relevant features for the model, we performed a feature selection process for numerical variables based on the results of logistic regression analysis. We also reduced the dimensionality of the variables using principal components analysis, with the maxi-mum explained variance criterion set at 75%. Finally, we selected the model with the best performance indicators based on the results obtained from the above process.

### 3.5. Screening for important predictive factors

In univariate analysis, there were statistically significant differences among temperature, formula feeding and GDM (*P* < 0.05) (Table 1). In previous studies, some factors including birth weight, GA, infant sex, Apgar score at 5 min, and maternal age also were reported to be closely associated with the development of NEC. Therefore, Multivariate analyses included factors with statistical significance and clinical significance that were identified by univariate analysis. In addition to factors that were statistically significant in the univariate analysis, clinically significant factors that did not reach the threshold of statistical significance were included in the multivariate analysis. This comprehensive approach allowed for a more thorough examination of all potential predictors of NEC in preterm and very low birth weight infants. The multivariable analysis results are shown in Table 2. Temperature (*P* = 0.003), Apgar score at 5 min (*P* = 0.004), formula feeding (*P* = 0.007) and GDM (*P* = 0.033) in the final model were statistically significantly correlated to NEC.

**Table 2:** Multivariable logistic regression analysis of risk of NEC.

Variables	OR (95% CI)	*P*
Maternal age	0.99 (0.92–1.06)	0.696
Birth weight	1.001 (0.99–1.003)	0.290
GA	1.07 (0.83–1.37)	0.785
Temperature	0.37 (0.20–0.73)	0.003*
Sex		
Male	0.56 (0.25–1.25)	0.157
Female	1	
Apgar score at 5 min		
0–7 points	6.90 (1.89–25.26)	0.004*
8–10 points	1	
Formula feeding		
No	1	
Yes	3.45 (1.40–8.49)	0.007*
GDM		
No	1	
Yes	2.35 (1.07–5.18)	0.033*

GA, gestational age; GDM, gestational diabetes mellitus; OR, odds ratio; CI, confidence interval.

* *P* < 0.05.

### 3.6. ROC plotting

**Figure 6:**
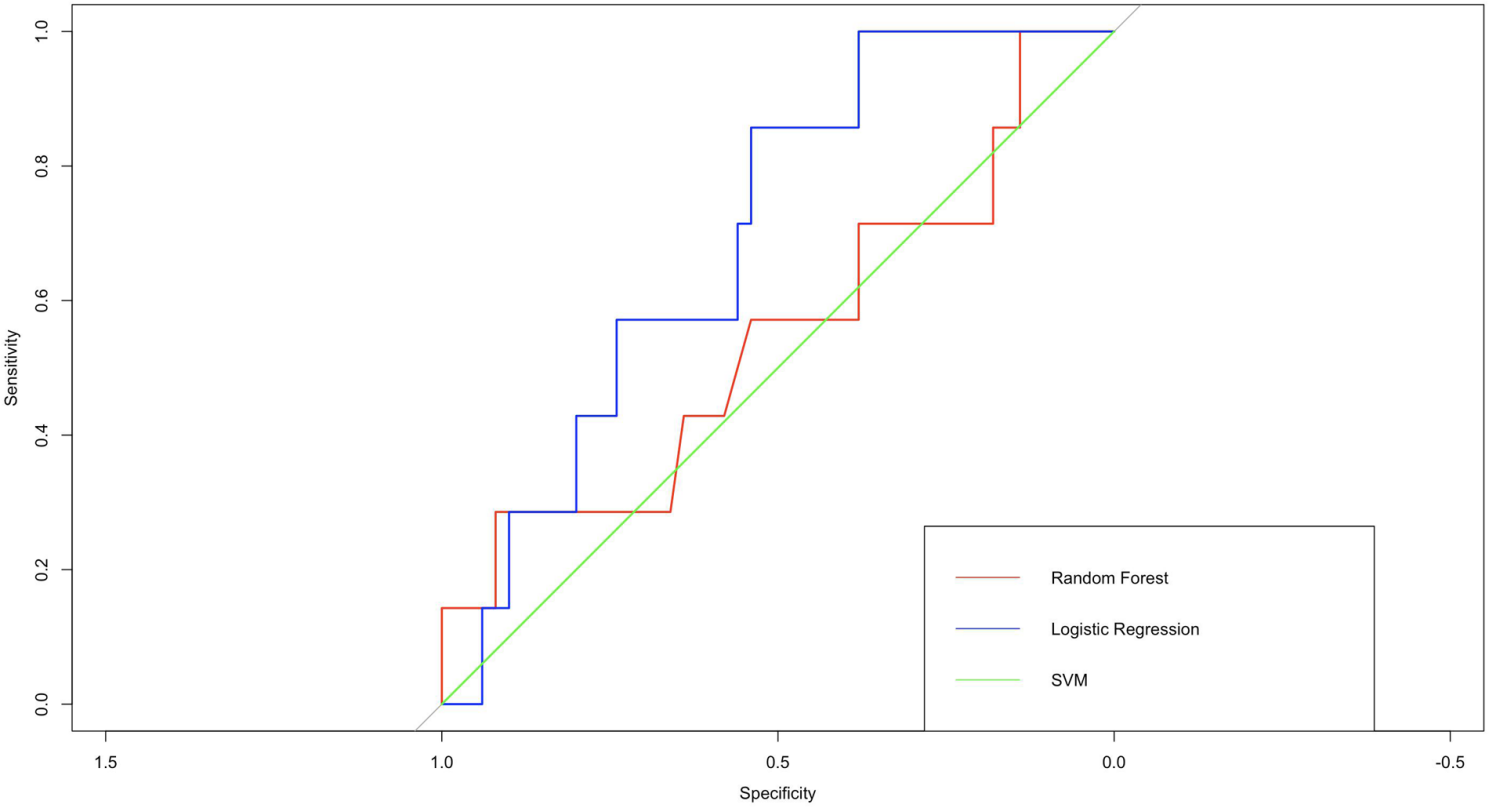
ROC curves of 3 models. In terms of AUC, logistic regression perform better than support vector machines and random forest.

To evaluate the performance of the logistic regression model, we created a ROC curve based on the actual and predicted classifications of the training set. The curve was created to determine the ability of the model to discriminate the presence of NEC in preterm infants, where 1 represents a positive classification and 0 represents a negative classification. Figure 6 shows that the AUC for the test set was 0.70, indicating good predictive accuracy and interpretability of the model.

The logistic regression model performed better than other models such as random forest and support vector machine in terms of prediction accuracy, AUC and other performance metrics. These results demonstrate the potential of the logistic regression model as a reliable and effective tool for predicting NEC in preterm and very low birth weight infants.

### 3.7. Risk prediction

**Figure 7:**
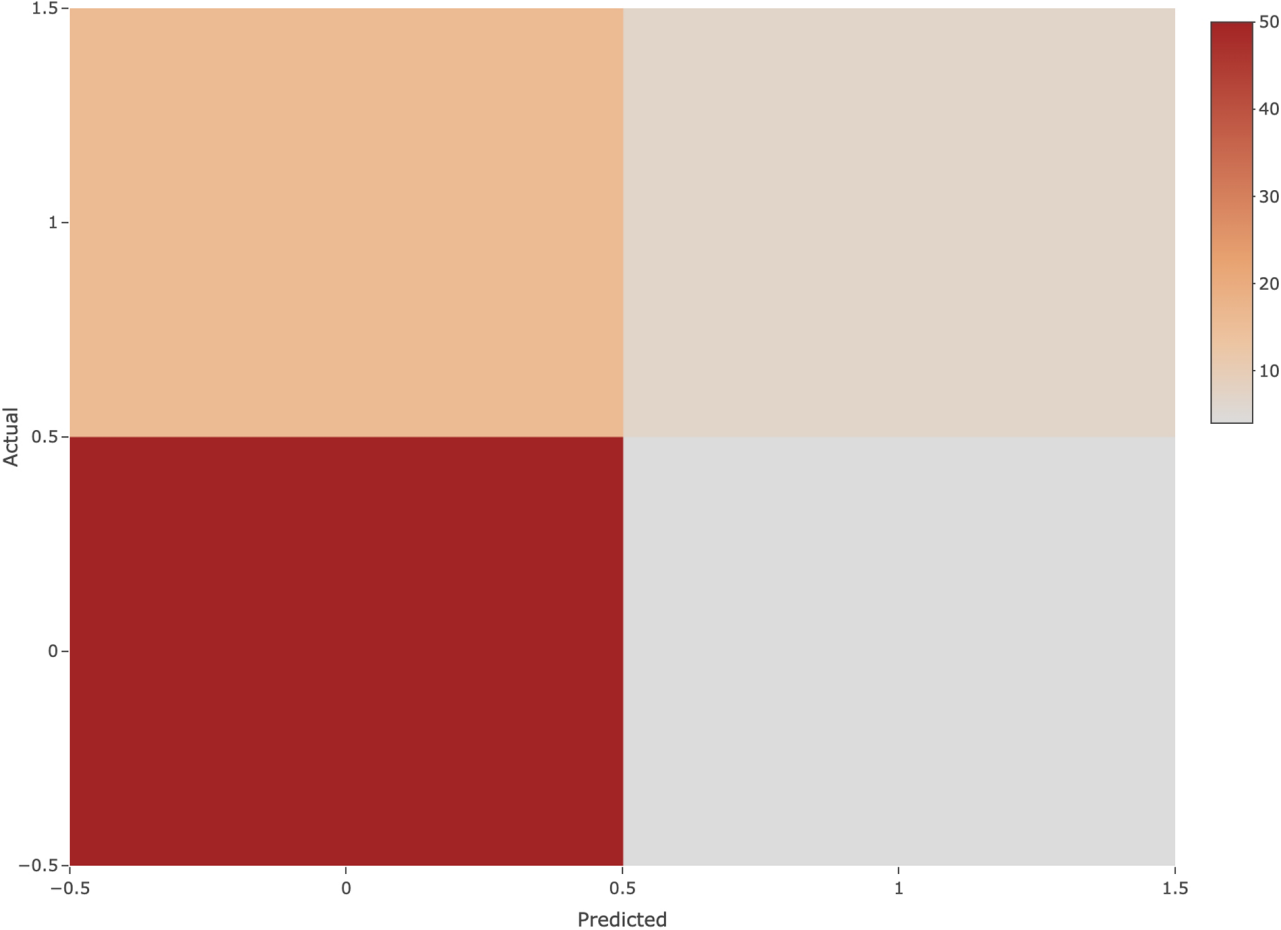
Confusion matrix. The horizontal axis indicates the predicted class, the vertical axis indicates the true class, 1 indicates the positive class, and 0 indicates the negative class.

The logistic regression model built using the above parameters achieved an accuracy of 82.46% on the test set with an area under the curve of 0.70. Analysis of the mixture matrix also showed that the model has good predictive performance. These results suggest that the prediction model has great potential to aid in clinical decision making, as shown in Figure 7.

Furthermore, it is important to note that logistic regression provides a probability estimate of NEC occurrence, ranging from 0 to 1. The model classifies samples with a probability less than or equal to 0.5 as negative predictions, indicating that those samples are not predicted to have NEC. Conversely, samples with a probability greater than 0.5 receive positive predictions, signifying the likelihood of NEC. Both Accuracy and AUC calculations consider the combination of predicted and true outcomes.

### 3.8. Compare with other models

The logistic regression model ranked first in terms of accuracy and AUC when compared to the results of support vector machines and random forests. This result confirms the choice of logistic regression as the appropriate classifier for this study, as shown in Table 3.

**Table 3:** Compare with other models.

Model	AUC	F1 score	Accuracy
Multivariate logistic regression model	0.70	0.90	0.82
Radom forest	0.54	0.93	0.82
Support vector machine	0.50	0.93	0.82

AUC, area under curve.

## 4. Discussion

Our study developed a model for predicting the occurrence of NEC using potential risk factors. This study revealed that Apgar score at 5 min, temperature, formula feeding and GDM were more important predictors of NEC among infants born <32 weeks of gestation and whose birth weight is <1,500 g. Good results and interpretability were shown by the ROC curve on the test set. The accuracy and AUC for the test set were 82.46% and 0.70, respectively. We external validated the model and found it to have high accuracy and robustness.

In the present study, Apgar score at 5 min between 0 points and 7 points was considered an important predictor of NEC. Previous studies have shown that an Apgar score of less than 7 points is one of the most important indicators for the diagnosis of neonatal asphyxia and is an important risk factor for neonatal asphyxia (19–21). According to the World Health Organization, it is estimated that around 20% of neonatal deaths worldwide occur as a result of asphyxia each year, with the rate rising to 60% in preterm infants, of which 6% may result in NEC (22). The essence of asphyxia is hypoxia (23). Infants with asphyxia are prone to defensive reflex blood redistribution, decreased mesenteric blood flow and intestinal mucosal ischemia and necrosis, which contributes to the development of NEC (24, 25). To conclude, our study underscores the potential importance of Apgar scores as NEC predictors in preterm and very low birth weight infants. However, the exact relationship between Apgar scores and NEC remains unclear. Further research involving larger cohorts and rigorous methodologies is necessary to validate this association.

Newborns have a lower efficiency in regulating their body temperature compared to adults, and are prone to both hypothermia and hyperthermia. These conditions can affect the growth and health of newborns. Previous studies have reported that hypothermia in low birth weight premature infants is significantly associated with increased in-hospital mortality and NEC (26–29). Similarly, our study also found a negative correlation between the body temperature of infants entering the NICU within 24 h after birth and the incidence of NEC, that is, the lower the body temperature of the infant, the greater the probability of developing NEC. When an infant's body temperature is too low within 24 h after birth, it should attract the attention of medical staff. However, it should be noted that the association between lower body temperature at admission and major neonatal diseases has not been fully established and more research is needed in this area.

As we know, GDM is a common pregnancy complication linked to adverse newborn outcomes (30, 31). The relationship between GDM and NEC has not been fully established. However, previous studies have shown that maternal gestational diabetes during pregnancy may increase the risk of NEC in their offspring (32, 33). The results of this study also support this view. Our research found that GDM was one of the independent risk factors for the development of NEC and has been included in the nomogram prediction model. This is not difficult to understand. Previous studies suggested that the mother is the nutrient supplier to the fetus, and high blood glucose during pregnancy can affect the intestinal blood flow to the fetus, leading to ischemic damage to the intestinal mucosa and thus increasing the risk of NEC (34).

Formula feeding was also an independent risk factor for NEC in our study. This is consistent with previous research (35). Chen et al. found that formula feeding was strongly associated with intestinal ischemia and hypoxia in human NEC (30). Meanwhile, according to data from animal experiments, formula feeding may lead to oxygen consumption exceeding its supply in pups, increasing the risk of intestinal hypoxia and promoting the development of NEC (36). Breast milk was the best food for newborns as it contained various immune components that could protect them from infections. Among these, Immunoglobulin A (IgA) and Immunoglobulin G (IgG) were the two most important antibodies that could neutralize pathogens in the intestines and blood, respectively. Additionally, breast milk contained active lymphocytes, macrophages, and specific antibodies that could recognize and eliminate various microorganisms. These immune components could effectively prevent serious intestinal diseases such as necrotizing enterocolitis (NEC) in newborns (37, 38).

Intestinal flora imbalance is recognized as a significant factor contributing to NEC development (39, 40). Consequently, there is a growing trend in early probiotic supplementation to modulate the intestinal flora (39, 40). However, in our study, we did not find a statistically significant impact of probiotics on the occurrence of NEC. The existing literature presents divergent findings regarding the effectiveness of probiotics in NEC prevention. Some studies emphasize that early probiotic supplementation significantly diminishes the incidence of NEC (41, 42). However, conversely, other investigations have indicated that probiotic administration might heighten the risk of NEC in extremely premature infants (43). This contradictory observation may be associated with the challenge that probiotics may fail to effectively adhere to the intestinal walls of preterm infants (43). Furthermore, we observed a lack of standardized criteria concerning the duration, dosage, and specific strains of probiotics used. Notably, variations in these aspects were evident across different studies (44–47). In summary, comprehensive future research endeavors focusing on different probiotic strains, optimal timing of administration, and standardized dosage regimens are warranted. These efforts will yield more precise and tailored guidelines for mitigating the risk of NEC in preterm infants.

In this study, we used multiple logistic regression, combining clinical data within 24 h after birth and maternal perinatal data, to construct a clinical prediction model for NEC and compare it with other commonly used machine models (support vector machines, random forests). External validation showed that the prediction model had good accuracy and calibration. However, our study has the following limitations. First, only infants with GA <32 weeks and birth weight <1,500 g were included in our study. The reason for this is that infants with GA <32 weeks and birth weight <1,500 g are prone to NEC (17). Second, the data in this study came from a single center. This is not generalizable. For this reason, we will look for an external validation assessment in a multicenter study.

## 5. Conclusions

In conclusion, this study developed a clinical prediction model using logistic regression to estimate the risk of neonatal necrotizing enterocolitis (NEC) in preterm and very low birth weight infants. The model demonstrated outstanding predictive performance in external validation. Key predictors included temperature, Apgar score at 5 min, formula feeding, and a history of gestational diabetes mellitus (GDM). This study provides a vital tool for predicting NEC risk in preterm and very low birth weight infants and is expected to enhance early clinical interventions, ultimately improving the survival and quality of life of affected children. However, further validation and additional studies are warranted to ensure the feasibility of its clinical application.

## Data Availability

The original contributions presented in the study are included in the article/supplementary material, further inquiries can be directed to the corresponding author.
